# Social network analysis - centrality parameters and individual network positions of agonistic behavior in pigs over three different age levels

**DOI:** 10.1186/s40064-015-0963-1

**Published:** 2015-04-17

**Authors:** Kathrin Büttner, Katharina Scheffler, Irena Czycholl, Joachim Krieter

**Affiliations:** Institute of Animal Breeding and Husbandry, Christian-Albrechts-University, Olshausenstr. 40, D-24098 Kiel, Germany

**Keywords:** Agonistic behavior, Pigs, Social network analysis, Centrality parameters

## Abstract

Knowledge of the network structure of agonistic interactions helps to understand the formation and the development of aggressive behavior. Therefore, video observation data of 149 pigs over three different age levels were investigated for 2 days each directly after mixing (65 groups in the rearing area, 24 groups in the growing stable and 12 groups in the breeding stable). The aim of the study was to use network analysis to investigate the development of individual network positions of specific animals and to determine whether centrality parameters in previous mixing situations have an impact on the future behavior of the animals. The results of the weighted degree centrality indicated that weaned pigs had a higher fighting intensity directly after mixing compared to growing pigs and gilts. Also, the number of different opponents (degree centrality) was higher compared to the older age groups. The betweenness centrality showed relatively small values and no significant differences between the different age levels, whereas the closeness centrality showed high values at all observed age levels. Experiences gained in previous agonistic interactions had an impact on the centrality parameters in subsequent mixing situations. It was shown that the position of individual animals in agonistic interaction networks can be characterized using social network analysis and that changes over different age levels can be detected. Therefore, social network analysis provides insights into the formation and evolution of behavioral patterns which could be of particular interest for the identification of key factors with regard to abnormal behavior (e.g. tail biting).

## Background

The mixing of unacquainted pigs, which leads to an unstable social structure, is a standard procedure in commercial pig production. An increase in agonistic interactions during the first few days after rehousing and mixing can be observed while the animals attempt to establish a new rank order. This negatively influences the animals’ health, welfare aspects and production parameters. A deeper understanding of how individual animals behave in this specific situation and how this behavior may change over time could be used for managing aggression and to implement or improve strategies for the reduction of agonistic interactions (Makagon et al. 2012).

This becomes even more important due to the fact that captive farm animals are housed in an artificial environment with restrictions made by humans. For instance, the animals have only limited space available with no or only few hiding-places or predetermined pen mates. Therefore, farm animals are to a lesser extent able to perform naturally or to follow behavioral rules in order to establish a stable group structure. Koene and Ipema (2014) confirmed this statement: farm animals are kept without regard of their inherent social behavior and rules, which can lead to increased agonistic interactions or behavioral problems.

One possible evaluation approach is to characterize the social structures with the help of network analysis. In a social network, the animals are illustrated as nodes and the connections between them, such as agonistic interactions, grooming or food competitions, are represented as the links of the network. These links can be directed, meaning each interaction has a clear initiator and receiver (e.g. attacker and victim) or undirected if the interaction has no clear orientation (e.g. sharing the same local area). Additionally, the interaction frequency between the same two animals can be considered as so-called weighted links. Unweighted or binary links do not contain additional information, they are either present or absent (Croft et al. 2011; Wasserman and Faust 1994; Wey et al. 2008). Social network analysis provides standardized mathematical methods to calculate network and centrality parameters (Newman 2010; Wasserman and Faust 1994). Furthermore, it offers a number of advantages to understand group structure and behavioral development in animal societies. Social network analysis treats animals as interdependent elements all connected to a network, accounting for the fact that the behavior of one animal in a group affects the behavior of its conspecifics (Asher et al. 2009). Social network analysis provides measures ranging from the characterization of the individual’s position in the network to global descriptors of the entire network (Krause et al. 2007). It can be used to determine groups and subgroups within a specific population and it can examine the interactions within and between these groups (Wolf et al. 2007). Furthermore, the temporal evolution of group structures can be analyzed (Drewe et al. 2009). At the individual level, social network analysis helps to range the animals in their central position within the group and thus to identify key animals (e.g. an individual which initiated the most agonistic interactions with other animals has the highest out-degree value in the group). Makagon et al. (2012) stated that the ability to improve the understanding of complex social structures by quantifying the social networks of animal groups and identifying the social roles of individual group members has important implications for applied animal behavior and welfare research.

Although it has been suggested that with the social network approach new insights into the formation and evolution of animal behavior with respect to management and welfare aspects can be gained, only a few studies have been carried out using this approach mainly focusing on wild or zoo animals (Lusseau and Newman 2004; Croft et al. 2005; Manno 2008; McCowan et al. 2008; Madden et al. 2009, 2011; Hinton et al. 2013). Especially, the number of studies which deals with social network analysis of captive farm animals is underrepresented.

The aim of this study was to analyze how the network position of specific animals develop over the three age levels under investigation (weaned pigs, growing pigs and gilts) and to determine whether the centrality parameters in previous rehousing and mixing situations have an impact on the future behavior of the animals. By quantifying the important aspects of the position of individual animals, information based on social network analysis could help to understand the formation and evolution of behavioral patterns, such as agonistic interactions. Furthermore, key factors for abnormal behavior, such as tail biting, could be identified with this additional information.

## Materials and methods

### Animals and housing

In the observation period from December 2010 till August 2012, video observation data of pigs at three different age levels (weaned pigs, growing pigs and gilts) were recorded on the “Hohenschulen” research farm of the Institute of Animal Breeding and Husbandry of the University of Kiel (Germany). The herd consisted of purebred and crossbred animals of the German Landrace and Large White breeds.

#### Weaned pigs

The research farm has four compartments with 10 flatdeck pens each. Each flatdeck pen measured 2.05 m × 1.36 m and had a concrete and metal base floor without substrate. After mixing and sorting by the smallest level of familiarity and by nearly equal weight, 6 to 11 weaned pigs were housed in each pen for about six weeks. Smallest level of familiarity means that the animals were sorted in such a way that they knew each other from previous mixings as little as possible. In the flatdeck pens, no animal was acquainted from the farrowing pens. According to the German norm (GfE 2006), the animals were fed ad libitum with solid pelleted feed and had access to two nipple drinkers for non-stop use.

#### Growing pigs

After the flatdeck period, the growing pigs were rehoused and mixed in groups of 20 to 25 animals in the growing stable. Similar to the mixing procedure in the flatdeck pens, the pigs were sorted based on equal body size and to minimize familiarity within the pen. Therefore, a maximum number of two animals acquainted with each other from the flatdeck pens were housed together. The dimension of the pens was 3.25 m × 2.40 m with a half-slatted and half-solid floor. In the growing stable, the animals were fed by an automatic mash feeding machine with a commercial diet (GfE 2006) and had ad libitum access to water which was accessible through nipple drinkers.

#### Gilts

In the 22nd week of age, the gilts were moved to the breeding stable. The groups of 18 to 29 animals were sorted to minimize familiarity within the pen, which means a maximum of five out of all pen mates were already acquainted from the growing pens. The breeding stable measured 7.20 m × 5.40 m and had a half-slatted and half-solid floor. In accordance to the German norm (GfE 2006), the gilts were fed by an automatic mash feeding machine with gilt feed. Water was accessible through nipple drinkers.

#### Ethical statement

The authors declare that the experiments were carried out strictly following international animal welfare guidelines. The institution the authors are affiliated with does not have research ethic committees or review boards (in consultation with the animal welfare officer of the Christian-Albrechts-University, Kiel, Germany). Therefore, the “German Animal Welfare Act” (German designation: TierSchG), the “German Order for the Protection of Animals used for Experimental Purposes and other Scientific Purposes (German designation: TierSchVersV) and the “German Order for the Protection of Production Animals used for Farming Purposes and other Animals kept for the Production of Animal Products” (German designation: TierSchNutztV) were applied. No pain, suffering or injury was inflicted on the animals during the experiments.

### Video observation data and behavioral measures

In the present study, data of agonistic interactions were recorded on video. The video observation started (at 12:00 h) and recorded the behavior of the animals directly after rehousing and mixing in the flatdeck pens, the growing and the breeding stable. Markings on the backs of the animals enabled the individual identification of the animals. Previous studies have shown that there was a decline in fighting behavior during the night (Stukenborg et al. 2010) and that the agonistic interactions fundamentally decrease after two days (Meese and Ewbank 1973). Therefore, the behavioral data were recorded for two days after rehousing and mixing excluding the period from 18:00 h to 07:00 h. The period used for analysis was limited to 17 hours in total (1st day: 12:00 h – 18:00 h; 2nd day: 07:00 h – 18:00 h). The HeitelPlayer software (Xtralis Headquarter D-A-CH, HeiTel Digital Video GmbH, Kiel, Germany) was used for the video analysis of the agonistic interactions. A total of 7,020 agonistic interactions between 1,354 animals were observed, whereby 149 individual animals were tracked the whole period from the age level weaned pig to gilt. The other animals were resold or brought to slaughter. These 149 animals were distributed in 65 groups in the rearing area (flatdeck), 24 groups in the growing stable and 12 groups in the breeding stable.

The start of an agonistic interaction is defined as an aggressive physical contact by one pig towards another which lasts longer than one second. These aggressive physical contacts can be ‘head to head knocks’ and ‘head to body knocks’, ‘parallel or inverse parallel pressings’, ‘bitings’ or ‘physical displacements’ (Tuchscherer et al. 1998; Puppe 1998; Stukenborg et al. 2012; Ismayilova et al. 2013). The agonistic interaction ends with a submissive behavior of an involved pig, i.e. turning away, displacement from a location or fleeing (Langbein and Puppe 2004; Tuchscherer et al. 1998; Stukenborg et al. 2012). The beginning of the agonistic interaction and the initiator or receiver of the fight were recorded. If the attacker or receiver could not clearly be identified, the fights were recorded with unclear starter and finisher (stand-off fights). In weaned pigs, the agonistic interactions were recorded by three different observers who had been trained at the beginning of the video analysis with the help of an unknown test sequence in order to practice the definition and the identification of the agonistic behavior. The inter-observer reliability was above 90%. The agonistic interactions of growing pigs and gilts were analyzed by only one person.

### Network construction

A social network of agonistic interactions was built for each pen. A link existed between two animals when they were both involved in an agonistic interaction. The networks were directed so that links were outgoing from the initiator of a fight and incoming to the receiver of a fight. Additionally, these links were weighted based on the contact frequency between two opponents in the aggregated networks. If the initiator or receiver of an agonistic interaction was unclear, two directed links were included in the network but with half of the initial weight assigned to it.

### Social network analysis

All calculations concerning the social network analysis were carried out using the Python module NetworkX (Hagberg et al. 2008).

#### Degree centrality

The degree centrality measures how well-connected an animal is, i.e. how many direct connections an individual has with others. Animals with a high degree centrality have agonistic interactions with many other individuals in the pen. Taking the direction of the links into consideration, i.e. it is known which of the animals is the initiator and the receiver of a fight, it is necessary to distinguish between the out-degree and the in-degree centrality (Newman 2010). A high value for the in-degree centrality means that the animal was attacked by many different pen mates, whereas a high value for the out-degree centrality means that this animal started fights with many of its conspecifics. In contrast, in unweighted relationships (binary), the occurrence of an agonistic interaction between any pair of animals determines the presence of a link, that is to say individuals which fight against each other are also linked in the network. In weighted relationships, the frequency of connections between two specific animals is additionally taken into consideration, implying that the number of agonistic interactions between a pair of animals defines the strength of the link (Madden et al. 2009).

#### Betweenness centrality

The betweenness centrality measures the extent to which an animal lies on the shortest paths between other individuals of a group. Animals with a high betweenness centrality are important for controlling the social connections within a group, especially if they serve as a bridge or cutpoint between two network components (Wasserman and Faust 1994; Freeman 1979; Croft et al. 2008). By removing these individuals, the connection between the two components is disrupted and the network can decompose into fragments (Newman 2010; Lusseau and Newman 2004; Flack et al. 2006; McComb et al. 2001). Thus, the identification of these cutpoints may have implications in animal management, as the removal of individuals with a high betweenness centrality from the group is likely to positively or negatively impact the social structure and the stability within the group depending on the analyzed interaction (Makagon et al. 2012).

#### Closeness centrality

The closeness centrality is based on the inverse of the shortest path lengths between a focal animal and all other animals in the network. In other words, the measure focuses on how close an individual is to all the other individuals in the network (Wasserman and Faust 1994). This measure takes not only the direct connections into account such as the degree centrality, but also the indirect interactions. Therefore, the interactions of individuals with a high value for the closeness centrality combined with a low value for the degree centrality could have a lot of indirect effects on the behavior of the other individuals in the network. Considering the direction of the connections, it is necessary to distinguish between the ingoing and the outgoing closeness centrality. In agonistic interaction networks, fights initiated by individuals with a high outgoing closeness centrality are passed in only a few steps to all other animals in the network, i.e. these animals influence all other pen mates in their own agonistic behavior by starting a fighting cascade. A high ingoing closeness centrality means that the focal individual is reached directly and indirectly by agonistic interactions of all other animals in the group in only a few steps. Therefore, a lot of fighting interactions end up at these animals.

#### Standardization of the centrality parameters

Both, the betweenness centrality and the closeness centrality are normalized by the number of nodes, so that the maximum value equals unity which enables a comparison of the values across networks of different sizes. The standardized measures range between 0 and 1 (Wasserman and Faust 1994). For the degree centrality, both the standardized and the unstandardized values were calculated. However, due to the fact that the values for both calculations did not substantially differ from each other and to enhance the comprehensibility, the values of the unstandardized degree centrality were used in the following.

### Statistical analysis

Statistical analysis was performed using SAS® statistical software package (SAS® Institute Inc 2008). Due to the small sample size in each pen and the distribution of the data, a Kruskal-Wallis-Test was used to assess the differences between the centrality parameters across the three observed age levels. Furthermore, a Spearman rank correlation was performed to characterize the relations between the centrality parameters.

## Results

### Network visualizations and timelines

**Figure 1:**
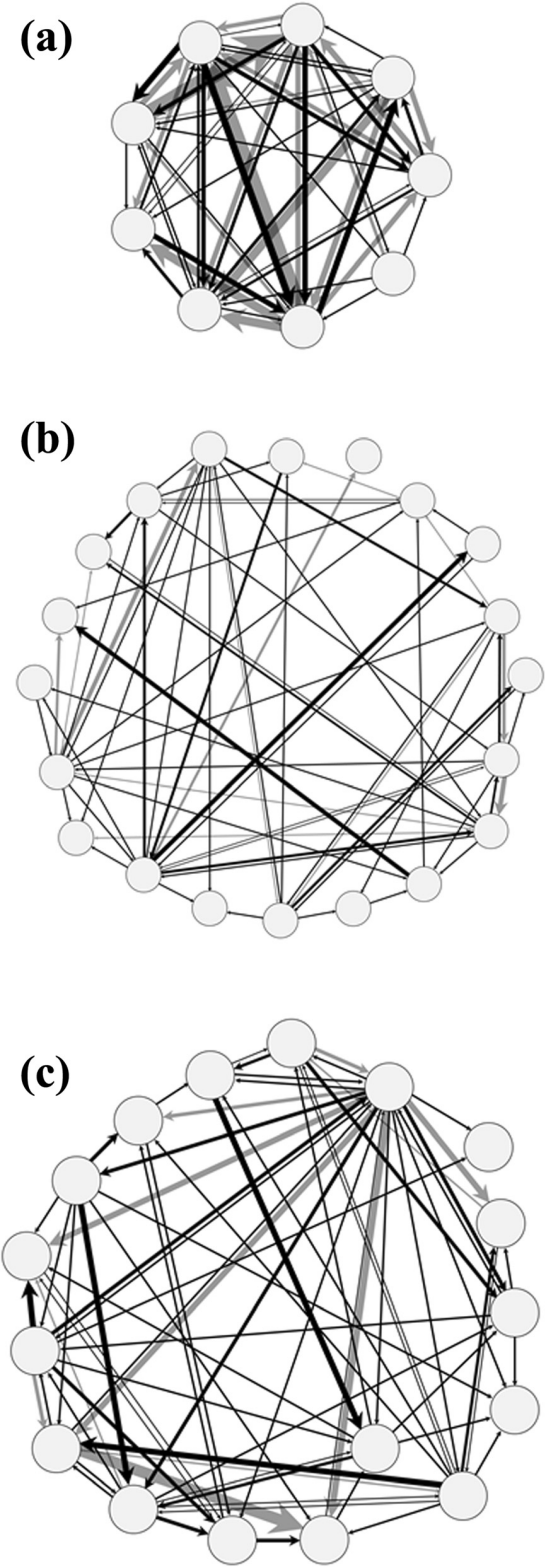
Agonistic interaction networks. One example for each age group: weaned pigs **(a)**, growing pigs **(b)** and gilts **(c)**. Each node represents an individual and each link represents an agonistic interaction. The frequencies of the interactions between two opponents are illustrated as the thickness of the connection. The shade of the links is only for reasons of clarity.

**Figure 2:**
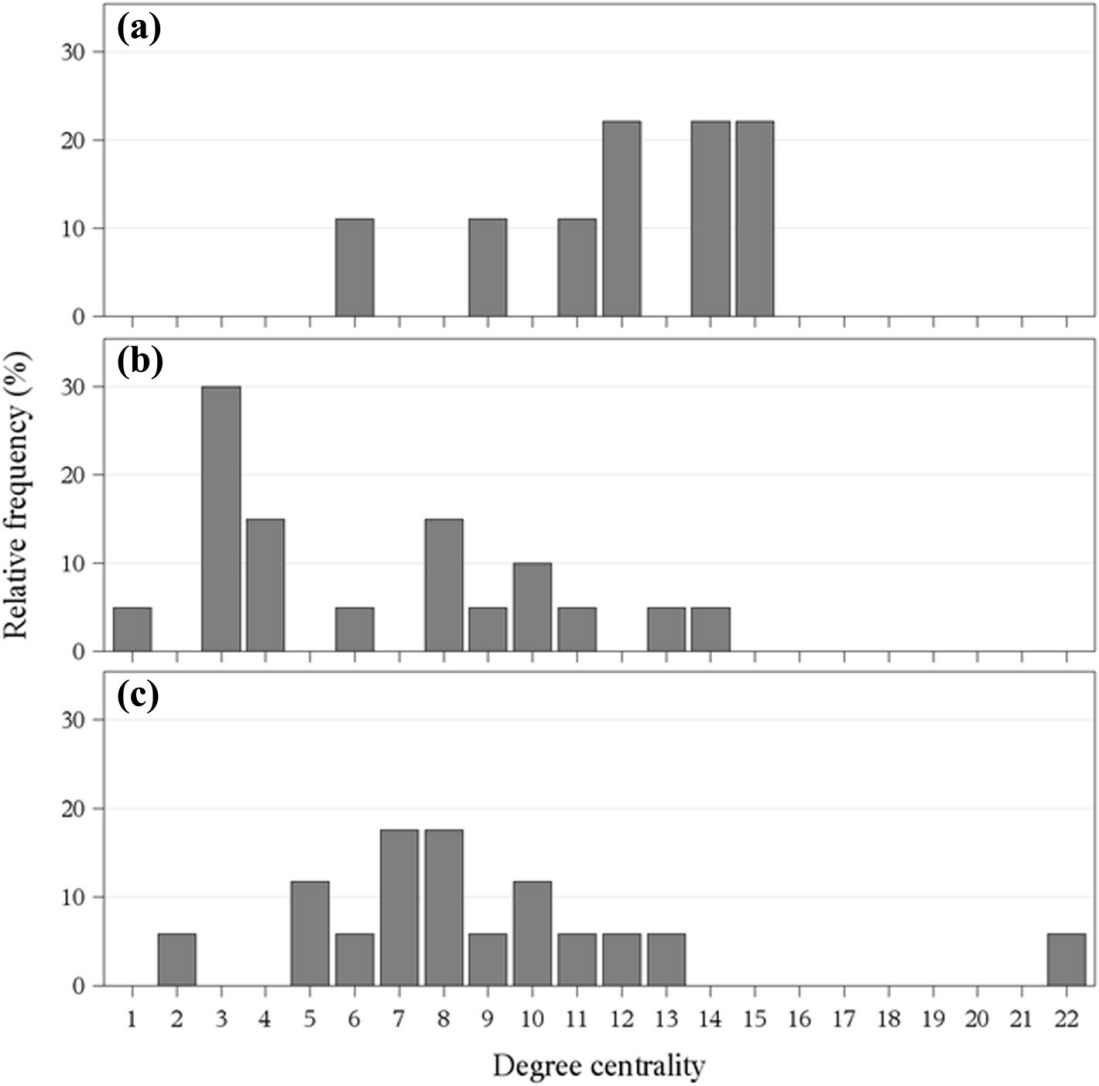
Degree distribution of the agonistic interaction networks. One example for each age group: weaned pigs **(a)**, growing pigs **(b)** and gilts **(c)**.

In Figure 1 an example of the agonistic interaction networks and in Figure 2 the corresponding degree distributions of weaned pigs (Figures 1a and 2a), growing pigs (Figures 1b and 2b) and gilts (Figures 1c and 2c) are illustrated. The network visualizations show the weighted networks aggregated over the observation period. In the example network of weaned pigs, an average in- and out-degree of 6 each could be obtained, i.e. average number of victims and attackers. The number of received fights varied from 1 to 8, whereas the number of initiated fights varied between 3 and 7. The number of agonistic interactions was 28 taking into consideration the interaction frequencies between the same opponents. This means that the animals in this specific flatdeck pen were involved in 28 fights in which on average 6 different animals were the victim or the attacker of the agonistic interaction, respectively. The animals in the growing stable had on average 8 agonistic interactions with 3 different victims or attackers each. The number of received fights varied between 1 and 5, whereas the number of initiated fights ranged from 0 to 10. The gilts in the breeding stable were involved on average in 12 fights with 4 different victims or attackers. Here, the number of received fights varied from 1 to 6 and the number of initiated fights ranged between 0 and 16.

**Figure 3:**
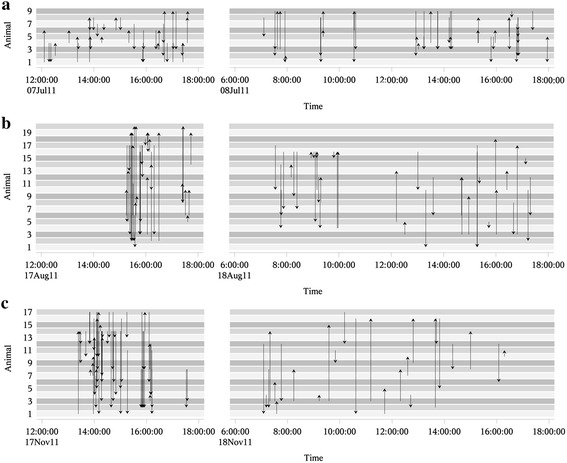
Timeline of the agonistic interactions. One example for each age group: weaned pigs **(a)**, growing pigs **(b)** and gilts **(c)**. Each grey-shaded stripe illustrates an animal. If an animal attacks another animal, an arrow is drawn from the attacker towards the victim. The numbers of the animals are random and do not indicate the same individuals in the three age levels.

Figure 3 illustrates the timelines of the agonistic interactions for the three example networks. Here, it can be seen that the weaned pigs had nearly evenly distributed agonistic interactions over the whole observation period, whereas in growing pigs and gilts, a decrease in fighting was observed after the first six hours after rehousing and mixing.

### Centrality parameters

Table 1 illustrates the results for the calculated centrality parameters for weaned pigs, growing pigs and gilts. The results are described below.

**Table 1:** **Median (range) of the binary and weighted centrality parameters calculated for the different age levels (weaned pigs, growing pigs and gilts)**

	**Weaned pigs**	**Growing pigs**	**Gilts**
Number of animals	9 (6 to 11)	23 (20 to 25)	24 (18 to 29)
**Degree**			
Binary	7^a^ (0 to 16)	5^b^ (1 to 18)	5^b^ (0 to 22)
Weighted	12^a^ (0 to 96)	5^b^ (1 to 27)	5^b^ (0 to 36)
**In-degree**			
Binary	4^a^ (0 to 8)	3^b^ (0 to 11)	3^b^ (0 to 11)
Weighted	7^a^ (0 to 51)	3^b^ (0 to 16)	3^b^ (0 to 17)
**Out-degree**			
Binary	3^a^ (0 to 9)	2^b^ (0 to 10)	2^b^ (0 to 16)
Weighted	5^a^ (0 to 73)	3^b^ (0 to 17)	2^b^ (0 to 29)
**Betweenness**			
Binary	0.03^a^ (0 to 0.50)	0.02^a^ (0 to 0.26)	0.02^a^ (0 to 0.32)
Weighted	0.04^a^ (0 to 0.63)	0.03^a^ (0 to 0.32)	0.02^a^ (0 to 0.37)
**Closeness**			
Binary	0.73^a^ (0 to 1.00)	0.43^b^ (0.04 to 0.73)	0.43^b^ (0 to 0.94)
Weighted	1.15^a^ (0 to 6.19)	0.51^b^ (0.04 to 0.93)	0.49^b^ (0 to 1.22)
**Ingoing closeness**			
Binary	0.61^a^ (0 to 1.00)	0.29^b^ (0 to 0.55)	0.32^b^ (0 to 0.60)
Weighted	0.84^a^ (0 to 5.22)	0.31^b^ (0 to 0.66)	0.32^b^ (0 to 0.79)
**Outgoing closeness**			
Binary	0.62^a^ (0 to 1.00)	0.30^b^ (0 to 0.60)	0.32^b^ (0 to 0.94)
Weighted	0.83^a^ (0 to 6.80)	0.34^b^ (0 to 0.89)	0.32^b^ (0 to 1.33)

^a,b^Within row values with different letters are significantly different (Kruskal-Wallis-Test, p < 0.05).

#### Degree centrality

In weaned pigs, the median number of agonistic interactions an animal was involved in was 7 and ranged between 0 and 16. In growing pigs and gilts, the values for the degree centrality were significantly lower with a median number of 5 agonistic interactions per animal, although a slightly wider range could be observed at these age levels (Kruskal-Wallis-Test: X^2^(2) = 29.46, p < 0.0001).

If the interaction frequencies are taken into consideration, the median weighted degree centrality in weaned pigs was 12 and ranged from 0 to 96 agonistic interactions per animal. Here, also significantly higher values than in growing pigs and gilts could be obtained (Kruskal-Wallis-Test: X^2^(2) = 67.07, p < 0.0001). In these age groups the range was also lower with 1 to 27 in growing pigs and 0 to 36 in gilts.

Similar tendencies could be observed for the binary as well as the weighted in-degree and out-degree centrality, i.e. the median number of different attackers and victims or the median number of received and initiated fights, respectively. The weaned pigs showed significantly higher values than the growing pigs or gilts (Kruskal-Wallis-Test: in-degree: X^2^(2) = 33.02, p < 0.0001; out-degree: X^2^(2) = 18.94, p < 0.0001; weighted in-degree: X^2^(2) = 80.20, p < 0.0001; weighted out-degree: X^2^(2) = 33.70, p < 0.0001). In addition, as with the weighted degree centrality in weaned pigs, the weighted in-degree and out-degree centrality in this age group showed the widest range with 0 to 51 and 0 to 73, respectively.

#### Betweenness centrality

No significant difference between the age levels could be observed for the betweenness centrality (Kruskal-Wallis-Test: X^2^(2) = 5.82, p = 0.0546). Here, the median values ranged from 0.03 in weaned pigs to 0.02 in growing pigs and gilts. The values for the weighted betweenness centrality showed the same trend. Here, also no significant difference between the age groups could be obtained (Kruskal-Wallis-Test: X^2^(2) = 3.29, p = 0.1928).

#### Closeness centrality

In weaned pigs, the median values for the closeness centrality was 0.73 and ranged from 0 to 1. In growing pigs and gilts, significantly lower values than in weaned pigs could be obtained with 0.43 (Kruskal-Wallis-Test: X^2^(2) = 247.64, p < 0.0001). If the interaction frequencies were included as edge weights, the median weighted closeness centrality showed higher values in all three age groups than the binary calculated values but with the same trend (Kruskal-Wallis-Test: X^2^(2) = 229.51, p < 0.0001).

Similar tendencies could be observed for the ingoing and outgoing closeness centrality. The weaned pigs showed significantly higher values than the growing pigs and gilts (Kruskal-Wallis-Test: ingoing closeness centrality: X^2^(2) = 225.12, p < 0.001; outgoing closeness centrality: X^2^(2) = 161.53, p < 0.0001). As with the weighted closeness centrality, the weighted ingoing and outgoing closeness centrality in weaned pigs showed higher values than the values based on the binary calculation. For growing pigs and gilts, the binary and weighted values were almost the same, although a wider range could be observed for the weighted ingoing and outgoing closeness centrality.

### Spearman rank correlations between the different calculated centrality parameters

Table 2 shows the Spearman rank correlations between the unweighted and weighted centrality parameters all in the range of 0.80 to 0.97. Due to these highly positive and significant correlation coefficients, only the Spearman rank correlations between the unweighted centrality parameters were considered in the further analysis (Table 3).

**Table 2:** **Spearman rank correlation coefficients between the unweighted and weighted centrality parameters**

	**Weaned pigs**	**Growing pigs**	**Gilts**
**Degree centrality**			
Unweighted - weighted degree	0.94*	0.97*	0.96*
Unweighted - weighted in-degree	0.86*	0.94*	0.94*
Unweighted- weighted out-degree	0.95*	0.97*	0.96*
**Betweenness centrality**			
Unweighted - weighted betweenness	0.84*	0.97*	0.94*
**Closeness centrality**			
Unweighted - weighted closeness	0.80*	0.92*	0.91*
Unweighted - weighted ingoing closeness	0.86*	0.91*	0.91*
Unweighted - weighted outgoing closeness	0.92*	0.96*	0.95*

*p < 0.05.

**Table 3:** **Spearman rank correlations between the different unweighted centrality parameters for weaned pigs, growing pigs and gilts**

	**Weaned pigs**	**Growing pigs**	**Gilts**
In-degree - Out-degree	0.70*	0.48*	0.61*
**Degree - Betweenness**			
Degree - Betweenness	0.41*	0.78*	0.86*
In-degree - Betweenness	0.32*	0.57*	0.73*
Out-degree - Betweenness	0.41*	0.75*	0.82*
**Degree - Closeness**			
Degree - Closeness	0.93*	0.82*	0.85*
Degree - Ingoing closeness	0.89*	0.61*	0.75*
Degree - Outgoing closeness	0.92*	0.80*	0.77*
In-degree - Ingoing closeness	0.95*	0.81*	0.87*
Out-degree - Outgoing closeness	0.97*	0.89*	0.87*

*p < 0.05.

#### In-degree – Out-degree

Table 3 shows that the correlation coefficients between the unweighted in-degree and out-degree were higher for weaned pigs with 0.70 than for growing pigs with 0.48 and gilts with 0.61.

#### Degree, in-degree and out-degree – Betweenness

The correlation coefficient between the degree centrality and the betweenness centrality were lower for weaned pigs with 0.41 than for growing pigs with 0.78 and for gilts with 0.86. The correlation coefficients between the in-degree as well as the out-degree centrality and the betweenness centrality showed similar results. Here, the values obtained for weaned pigs were also smaller than for growing pigs and gilts. However, as the values for the correlations between the degree as well as the out-degree centrality and the betweenness centrality in growing pigs and gilts had nearly the same height, smaller values for the correlation coefficients of the in-degree centrality and the betweenness centrality for growing pigs with 0.57 could be observed.

#### Degree, in-degree and out-degree – Closeness, ingoing closeness and outgoing closeness

The correlation coefficient between the directed and undirected degree centralities and the closeness centralities showed highly positive values for all three observed age levels (Table 3). However, the correlation coefficients for the weaned pigs were slightly higher than for growing pigs and gilts.

### Spearman rank correlations of the centrality parameters between the different age levels

Table 4 shows the Spearman rank correlations of the binary and weighted centrality parameters between the three age levels. Only the correlations between consecutive age levels were significant. Therefore, the correlation coefficients between weaned pigs and gilts are not illustrated here.

**Table 4:** **Spearman rank correlations of the binary and weighted centrality parameters between the three age levels weaned pigs, growing pigs and gilts**

	**Binary**	**Weighted**
**Degree**		
Weaned pigs - Growing pigs	0.19*	0.23*
Growing pigs - Gilts	0.19*	0.23*
**In-degree**		
Weaned pigs - Growing pigs	−0.02	0.05
Growing pigs - Gilts	0.07	0.09
**Out-degree**		
Weaned pigs - Growing pigs	0.27*	0.33*
Growing pigs - Gilts	0.25*	0.28*
**Betweenness**		
Weaned pigs - Growing pigs	0.12	0.18*
Growing pigs - Gilts	0.02	0.04
**Closeness**		
Weaned pigs - Growing pigs	0.16	0.17*
Growing pigs - Gilts	0.30*	0.34*
**Ingoing closeness**		
Weaned pigs - Growing pigs	0.06	0.08
Growing pigs - Gilts	0.18*	0.14
**Outgoing closeness**		
Weaned pigs - Growing pigs	0.28*	0.31*
Growing pigs - Gilts	0.22*	0.19*

*p < 0.05.

#### Degree centrality

Low but positive and significant correlation coefficients between weaned pigs and growing pigs as well as between growing pigs and gilts could be obtained for the degree centrality. The values ranged between 0.19 and 0.23. Slightly higher and also significant correlations were carried out for the out-degree centrality, whereas for the in-degree centrality no significant correlations between the different age levels could be obtained.

#### Betweenness centrality

A low but significant correlation of the weighted betweenness centrality was found between weaned pigs and growing pigs with a correlation coefficient of 0.18. The other combinations showed no significant results.

#### Closeness centrality

The correlation of the binary closeness centrality for the comparison between weaned pigs and growing pigs showed no significance. All other possible combinations were positively and significantly correlated with each other, while the correlation between weaned pigs and growing pigs (weighted calculation) showed a smaller value with 0.17 than the correlation between growing pigs and gilts with 0.30 for the binary calculation and 0.34 for the weighted calculation. Significant and positive correlations could be obtained for the outgoing closeness centrality, which ranged from 0.19 to 0.31. For the ingoing closeness centrality, only the correlation between growing pigs and gilts was significant (binary calculation).

## Discussion

### Network visualizations and timelines

Figure 1 illustrates the three weighted agonistic interaction networks of weaned pigs, growing pigs and gilts aggregated over the whole observation period. Compared to the timelines in Figure 3, this implies an abstraction of the real fighting activities which can fluctuate within time. Especially the two network examples of growing pigs and gilts showed more agonistic interactions in the first six hours after rehousing and mixing. This information was lost in the aggregated networks (Blonder et al. 2012). However, due to the relative short observation period, the analysis of these aggregated networks give an insight into the formation and evolution of agonistic interactions directly after rehousing and mixing and enables therefore the comparison of different age levels.

### Centrality parameters

Beside the unweighted centrality parameters, the weighted centrality parameters were analyzed in order to include the interaction frequencies of two specific opponents. Although other studies (Koene and Ipema 2014) stated that for small groups of animals with small datasets weighted social network analysis could probably be more powerful, the results of the present study showed high correlation coefficients between unweighted and weighted centrality parameters. Therefore, the further analysis focused on the unweighted centrality parameters. Whether the results of a weighted or an unweighted approach differ from each other, depends not exclusively on the group size, but also e.g. on the recorded interaction or the space allowance the animals have.

The results of the degree and the closeness centrality indicate that the weaned pigs fought more during the first two days after rehousing and mixing compared to growing pigs and gilts and could also reach their pen mates faster than the older age groups. Also, the number of different opponents was higher than in the older age groups, which can be derived from the results of the unweighted degree centrality. The relatively high values for both centrality parameters obtained in the flatdeck pens can be explained by the fact that nearly all animals in this age group fought against each other in the first two days after rehousing and mixing which led also to small distances between the single animals. In growing pigs and gilts, the number of fights decreased and a shift in the agonistic interactions towards specific animals could be observed. Therefore, lower values for the median closeness centrality but with a relatively high range could be obtained in these higher age levels. If agonistic interactions or other connections with a negative connotation are analyzed, it is necessary to consider that the outgoing centralities (e.g. out-degree and outgoing closeness centrality) measure the active behavior and the ingoing centralities (e.g. in-degree and ingoing closeness centrality) measure only the passive behavior. This is of particular importance when the social network structure of captive farm animals is analyzed. Here, the natural behavior of the animals is influenced by restrictions made by humans, e.g. limited space available and predetermined pen mates. For instance, a subordinate animal can avoid more easily an agonistic interaction in the wild, whereas in the artificial environment of a stable with limited space available only a low means of escape exists. Furthermore, the decrease in agonistic interactions with repeated rehousing and mixing situations can be explained as habituation effect (Coutellier et al. 2007). Alternatively, according to Hessing et al. (1993; 1994), the decrease could also represent a new coping strategy towards an unstable social structure in which the animals develop a preference for coping behaviors which are able to limit the energy costs and the number of injuries. As a result, a new stable social structure is established with fewer agonistic interactions (van Putten and Buré 1997). Due to the practical conditions, it has to be taken into account that the comparison between the different age levels could also be influenced by the different group sizes, by the available space (i.e. bigger pens with increasing age level) as well as by the increasing level of familiarity with higher age level. Marchant-Forde and Marchant-Forde (2005) stated that smaller group sizes in pigs do appear to have more post-mixing aggressions in comparison to larger group sizes. Other studies confirmed this statement (e.g. Nielsen et al. 1995; Andersen et al. 2004; Turner et al. 2001). However, also the pen size could be an explanation for this relation. The animals have in larger pens a greater distance available to avoid agonistic interactions, whereas in smaller pens they have to face them. Moreover, familiar pigs were engaged in fewer agonistic interactions than unfamiliar pigs (Puppe 1998). Also Arey and Franklin (1995) stated that the number of fights increased significantly with the number of unfamiliar animals. These findings could not be confirmed by Jensen and Yngvesson (1998) who did not find significant differences in the fighting behavior between unknown and already acquainted animals.

For the betweenness centrality low values and no significant differences between the three age levels could be obtained. This can be explained by the small group size and the limited space available. In larger groups, it is more likely that some animals form so-called bridges or cutpoints between different network components and as a consequence thereof these animals have a high betweenness centrality (Newman 2010).

### Spearman rank correlations between the different calculated centrality parameters

#### In-degree – out-degree

For weaned pigs, a higher correlation coefficient between the in-degree and the out-degree centrality than for growing pigs and gilts could be obtained. This difference can be explained by the more stable rank order of growing pigs and gilts due to their increased familiarity and their experiences acquired from previous agonistic interactions. According to D’Eath (2004) and Otten et al. (1997), previous experiences of success or failure in aggressive interactions have long-lasting effects on the animals. Here, the previous dominance rank in particular had a prolonged effect on the rank position in later groups.

In the present study, correlation coefficients of 0.48 to 0.70 could be obtained between in-degree and out-degree. According to Szell et al. (2010), the correlations between the in-degree and the out-degree centrality in networks based on positive links are almost balanced with a correlation coefficient close to 1, whereas lower correlation coefficients in networks with negative links can be observed, such as in agonistic interaction networks. Although the correlation coefficients obtained are still relatively high for an agonistic interaction network, this finding can be explained by the fact that only the first two days after rehousing and mixing were analyzed in which the rank order of the animals had not been completely established. It is expected that a further decrease in the correlation coefficients can be observed after the stabilization of the rank order.

#### Degree, in-degree and out-degree – Betweenness

All correlation coefficients between the degree centralities and the betweenness centrality showed smaller values for weaned pigs than for growing pigs and gilts. This can be explained by the fact that the rehousing and mixing procedure after the farrowing stable is the first situation of this kind for the animals. Due to the fact that the animals were mixed and sorted by the smallest level of familiarity, they did not know each other from the previous pen and, therefore, all animals were involved in agonistic interactions in order to establish a stable rank order. As seen above, one explanation for these results could be that growing pigs and gilts specifically chose their opponents due to their experiences from previous agonistic interactions and also due to their confidence in their own fighting ability, indicating that they had learned to weigh up their chances of winning a fight.

#### Degree, in-degree and out-degree – Closeness, ingoing closeness and outgoing closeness

In weaned pigs, the correlation coefficients between the degree centralities and the closeness centralities showed higher values than for growing pigs and gilts. The higher values in weaned pigs can be explained by the first rehousing and mixing situation and the lack of experiences from previous agonistic interactions. They needed more agonistic interactions to establish a stable rank order. However, the values in growing pigs and gilts were also medium to high correlated. In small networks, one can say that the higher the degree centrality of a specific animal, the smaller is the distance of this animal to its pen mates, which is therefore correlated with the closeness centrality.

According to Krause et al. (2015), a primary advantage of social network analysis over other analytical methods is the ability to quantify indirect relationships or associations which allows for the detection of complex social structures. However, in the present study high correlation coefficients between the degree and the closeness centrality could be observed, indicating that this advantage only becomes apparent for moderate to large groups or otherwise if enough space is available to express for example evasive or avoidance behavior.

### Spearman rank correlations of the centrality parameters between the different age levels

The relations between the centrality parameters over the observed age levels illustrate that only the consecutive age levels, i.e. weaned pigs to growing pigs and growing pigs to gilts, showed significant results. This indicates that experiences from previous rehousing and mixing situations significantly influence the behavior of the animals, which is in accordance with the findings described above that the animals gained confidence in the rank position already achieved (D’Eath 2004; Otten et al. 1997). Therefore, it is only possible to draw conclusions from one age level to the next, even though here only small correlation coefficients could be obtained.

For the correlations of the degree centrality over the different age levels, the out-degree centrality showed more stable results compared to the in-degree centrality. Similar results could be obtained for the outgoing closeness centrality. These findings can be explained by the fact that these centrality parameters are based on an active behavior, whereas the in-degree centrality depends on the aggression of the pen mates and the possibility to flee. The correlations of the betweenness centrality over the different age levels showed ambiguous results. Due to the small group size, the betweenness centrality only adopts relatively small values which can easily change with small changes in the network structure. Therefore, no clear trend could be observed.

### Possible applications of social network analysis for management and welfare issues

According to Hinton et al. (2013), social network parameters could prove important in revealing potential causes of animal stress. For instance, with the help of centrality parameters, animals which show a disproportional amount of aggression or abnormal behavior, such as tail biting, as well as their victims can be detected. On this basis, it could be investigated how the network structure changes if some key individuals (i.e. with high centrality values) are removed from the group (e.g. Lusseau 2003). Depending on the chosen centrality parameter, the development of the agonistic interactions and the general stress level in the group could be compared and the appropriate parameter can be chosen to realize a maximum calming within the group. This approach would also reveal important insights in the relationship between the group members. Furthermore, the winner and the looser as well as the duration of an agonistic interaction should be included in the analysis. For example, a high out-degree centrality does not necessarily imply that it has also won the majority of the fights. Moreover, one cause for a high betweenness centrality may be the fact that the animals won a lot of fights and thus gained self-confidence in their own fighting ability. Therefore, they initiated fights with other individuals in the group. Another possibility for a high betweenness centrality may be that these animals lost agonistic interactions and reflected their frustration by an attack towards animals with a lower rank position. The analysis of this kind of associations could be used to identify key factors triggering harmful behavior.

Beside these relations between the animals, also resources, such as the access to feed or enrichment material, can be included in the network analysis as so-called multi-partite networks (Wasserman and Faust 1994). Previous studies showed that the supply of enrichment material reduced the number of aggression in the pen (Beattie et al. 1996; Schaefer et al. 1990) and that the feeding system had a large impact on the aggression among the animals (Hansen et al. 1982; Vargas et al. 1987). With this approach the questions could be answered why animals show aggressive behavior and if this behavior is correlated with a refused access to feed or enrichment material and therefore with an increased level of frustration.

## Conclusion

The reduction of agonistic interactions is a common challenge in commercial pig production. It improves not only animal health and welfare aspects, but also production parameters, which are important from an economic point of view. The aim of the present study was to analyze how the network position of individual animals developed over three different age levels and to determine whether the former centrality parameters had an impact on subsequent rehousing and mixing situations. The results of the degree centrality indicated that weaned pigs have a higher fighting intensity directly after rehousing and mixing compared to growing pigs and gilts. The results of the unweighted degree centrality showed that the number of different opponents is also higher compared to the older age groups. The betweenness centrality showed relatively small values and no significant differences between the different age levels, whereas the closeness centrality showed high values at all observed age levels. In contrast to wildlife populations, the agonistic interactions of captive farm animals are highly influenced by restrictions made by humans, e.g. limited space available in each pen, predetermined pen mates and a lack of a means of escape. Due to these restrictions the animals are not able to perform natural behavioral patterns as in the wild. Furthermore, experiences from previous agonistic interactions have an impact on the centrality parameters in subsequent rehousing and mixing situations. The present study showed that social network analysis can be used to characterize the position of individual animals in agonistic interaction networks and to describe its change over different age levels. Therefore, new insights into the formation and evolution of behavioral patterns can be gained. Social network analysis provides diverse evaluation methods especially for the identification of key factors with regard to abnormal behavior, such as tail biting.
